# A Contribution to the Study of Hog Cholera

**Published:** 1878-03

**Authors:** 


A Contribution to the Study of Hog Cholera.—An invitation from the Columbus Academy of Medicine to read a paper on the disease familiarly known as hog cholera, should be accepted as a proof that physicians of this city are not indifferent to the malady which so seriously affects the prosperity of the surrounding country. It also affords evidence that physicians are not wanting in a humane desire to alleviate the sufferings of the creatures we subordinate to our pleasure or profit. It may be presumed that the members of the Academy are aware of the resemblances between some animal diseases and those which affect mankind; and of the facilities which animals afford for the study of such diseases. The communicability of some diseases from animals to men is equally well known to you, as well as the influence which the diseases of milk and flesh-producing animals may have upon the health of the people. Permit me to add, that in a country where well-educated veterinarians are very scarce, there would seem to be a necessity that physicians should care not only for the families of their patrons, but also for their four-footed dependants.

I regret that what I have to offer this evening is only "A Contribution to the Study of Hog Cholera," instead of an exhaustive treatment of the subject. My opportunities for investigation have been limited, and have occupied only such time as could be spared from the pressure of other duties. I have observed somewhat hastily several affected herds of swine, and examined the bodies of some fifty, supposed to have been subjects of this disease, several of which were killed in order to determine what structural changes were discoverable in the earlier stages. I have not had sufficient opportunity to test the comparative efficacy of different modes of treatment.

*History.*—So far as I am informed, a serious and often fatal disease of swine, known as hog cholera, has been more or less prevalent in Ohio for twenty years. Whether this disease is identical with any disease of swine of former periods or of other countries, is a question to which it may be presumed that sufficient attention has not been given, inasmuch as widely diverse opinions are entertained. Of late years the disease has presented itself in almost every State of the Union; but especially in States West and South. The report of the Department of Agriculture for

1876 shows a loss in the State of Missouri of thirty per cent. of all the swine in the State; in the States of Illinois and Kentucky the loss was more than twenty per cent.; in Indiana eighteen per cent.; in Georgia ten per cent.; and in Ohio seven per cent. The aggregate loss of the whole country was twenty millions of dollars.

*Symptoms.*—The first stage of the disease is congestive; the animal crawls under the litter, or huddles close to his companions, refuses solid food and appears sluggish; this stage may last a few hours or it may continue for a day or two. If the animal does not die appoplectic in the congestive state, reaction follows; then there is thirst, feverishness, and redness of the skin, especially where the hairy covering is least abundant. In some animals this redness is diffused, but more frequently it appears in spots, from which the cuticle is sometimes raised in small blisters; the conjunctiva is reddened and the mouth is dry. The pulse is from 100 to 120, and the temperature from 104° to 106°; and in one instance I found it as high as 111°. Soon the abdomen becomes tumid and tender, the animal expresses great pain when compelled to move, and prefers to lie prone upon the belly. There is often great difficulty of breathing, which is more or less spasmodic, and attended with severe cough; not unfrequently there is hemorrhage from the nostrils and swelling of the parotid glands. Through the febrile, or acute inflammatory stage, there is constipation, which is afterwards followed by diarrhœa; at first the evacuations may be destitute of bile, then bilious, and very soon dark colored, or blood-stained and very fœtid. Finally, locomotion becomes difficult, unsteady, and perhaps spasmodic, or the posterior extremities are entirely paralyzed. Death may occur within a few hours and during the congestive stage as already stated; it may occur in a few days from the effects of inflammation, or in one or two weeks from exhaustion, from peritonitis, or from effusion.

*Post mortem.*—On examination after death, the cavity of the abdomen is often found to contain a few ounces of serum, and sometimes fibrinous exudations. The liver is not materially changed in size or texture, but often has a yellowish or ashy color; occasionally it was found greatly engorged. The spleen was enlarged in nearly half the cases examined, sometimes to a great extent, and so disorganized that when the capsule was ruptured the grumous contents would escape. This disorganized condition of the spleen, as might be expected, was greatest when the blood showed the most change.

The stomach often contained undigested food, the mucous membrane was usually congested, but rarely showed as decided evidence of inflammation as other portions of the alimentary canal. The small intestines, on their external surface presented patches of deep discoloration, occasionally they would be glued together by lymphy exudations. The mucous lining showed general congestion, and inflammation in patches; sometimes tumefaction and exudation would be almost confined to the aggregate and solitary glands, while in some cases, where covered by dark colored crusts, or where these had sloughed off, patches of ulceration were seen. Frequently, however, the inflammation and ulceration affected glandular and other portions indifferently; or the whole inner surface of the bowel would be inflamed for several inches in extent. In the large intestines the mischief was still more apparent, the cœcum often contained a multitude of inflamed and elevated spots, or in their place ulcerations as large as the impressions made by the ends of one's fingers on a dusty table, and as close as the fingers held together could make them. The colon usually presented ulcerations similar to those of the cœcum; its contents were small masses of hardened fæces, dark colored or blood-stained; and not unfrequently dark fluid blood in considerable quantity. Sometimes portions of the colon were almost gangrenous; in a few cases the intestines had been perforated by ulceration and peritonitis had resulted. The mesenteric glands were almost always enlarged; the kidneys were congested, the bladder contained healthy looking urine, or it was tinged with bile or blood.

The lungs were always congested and often inflamed, or impervious to air from serous infiltrations; the air-passages were filled with frothy mucus, the trachea and larynx were sometimes found in a state bordering upon gangrene. In a few instances, where the cough had been an unusually marked symptom, the smaller air-passages were crowded with nematoid or thread-like worms, the strongylus paradoxus; these cases were so few that the lung-worms can only be regarded as an accidental complication.

Effusion within the pleura, or pericardium, was sometimes seen; the blood in the heart cavities was clotted, but the clots were soft and in general the blood elsewhere was fluid. On examination of the blood with the microscope, the blood-globules appeared shrunken and their margins irregular. In the blood drawn from some animals that were killed, bacteria were seen with a power of from eighty to one hundred diameters. The bacilla anthracis, which is said to be pathognomonic of anthrax fever, was looked for but not found. The brain and spinal marrow were repeatedly examined, but without disclosing any marked lesion.

*Pathology.*—That this disease corresponds in no degree with Asiatic cholera of the human subject, is evident on slight examination. In hog cholera constipation is much more common at the outset than diarrhœa, and afterwards when diarrhœa is present, the evacuations are unlike the characteristic discharges of Asiatic cholera. That the disease is an idiopathic fever of typhoid type, substantially like typhoid fever of the human subject, would seem to be proved by the congestion, inflammation and ulceration so generally found in the intestinal canal, by the frequency of hemorrhages from the bowels, and the frequent enlargement of the mesenteric glands and spleen; also by the petecchial eruption over the surface, and from its greater tendency to attack young animals. On the other hand, hog cholera resembles what is known in Europe as anthrax fever, the symptoms in living animal being almost identical, and the *post mortem* appearances not greatly dissimilar. Hog cholera differs from what are styled anthracoid diseases by its much less malignity; and, although in my opinion it is contagious so far as to be communicated in some way from diseased to healthy swine, it does not appear to be transmissable to other species of animals, nor to man, while the anthrax fever of Europe passes readily from one kind of stock to another and is often fatally communicated to mankind. The blood of animals suffering from anthrax fever, it is said, always contains the bacillus anthracis. So far as my examinations have gone or my information extends, the presence of bacillus has not been demonstrated in hog cholera. I should add, however, that many European veterinarians regard what is styled anthrax fever of swine as nothing more nor less than typhoid fever; but the frequency of intestinal lesions, and severe suffering in hog cholera, are adverse to such a conclusion.

*Cause.*—First, contagion. It seems to be established that this disease has been communicated to healthy swine by bringing them in contact with the affected, or by confining them in cars, pens or yards that diseased animals have recently occupied. It appears, also, that the contagion may, in some way, be conveyed for the distance of half a mile or more; also, that animals kept in the most cleanly and thrifty condition are not proof against the contagion. If the disease is typhoid fever, we should expect to find that contaminated water is a principal medium through which the disease germs pass from one animal to another. How far exemption from attack may be secured, when the food and drink are free from all possible contamination, has not, so far as I know, been determined. Second. It seems, also, to be established that healthy hogs, if subjected to hard driving for considerable distances in hot weather, or if crowded together in large numbers, may develope the disease *de novo;* and, possibly, other conditions and circumstances not yet understood, may tend to a similar result.

*Treatment.*—With this malady it is especially true that prevention is better than cure; the attempt to medicate a case of hog cholera, when, by so doing, there might be danger of exposing other animals, is the reverse of economy. When it is certain that an animal has this disease, it should be killed immediately and buried, and the whole premises thoroughly disinfected. For this purpose, all litter and rubbish should be burned, and the pens, or styes, fumigated with burning sulphur. Hogs that have been in contact with sick ones, may be expected to show symptoms of the disease, after thirteen or fourteen days, at furthest, if the weather be cold; if the weather should be very hot, the period of incubation may be shortened to three or four days. During the period of incubation, sulphur should daily be given with the food, or the hyposulphite of soda may be used instead. This is a cheap drug, and a sufficient quantity will be taken, without objection, if dissolved in the drink. Some farmers think they have kept off the disease, after exposure, by acidulating all the food slightly with sulphuric acid; others believe that the sulphate of iron is equally effectual. The carbolic, cresylic and salicylic acids may prove better disinfectants than those already named; or, possibly, change of quarters may be more beneficial than any plan of medication. If an attack of the disease cannot be prevented, and loss of appetite, with nausea, and constipation are noticed, then laxatives are clearly indicated, and sulphur enough to act may easily be given with the food or drink, or castor oil, with spirits of turpentine, may be administered to an animal that is conveniently small, and repeated, from time to time, so long as enteric inflammation continues. Corn, boiled in weak lye from wood ashes, is relied upon by some, while others place soft soap where the sick animals will take it, instinctively, to relieve the acidity of the stomach and bowels, from which all seem to suffer. After the disease has run its course, and left the animal without appetite, and with an exhausted and fœtid diarrhœa, the sulphate of iron answers a good purpose, combining tonic with astringent effects. How far such treatment would prove effectual, if intelligently followed, I am not prepared to say. I have several times been thanked for suggesting such a course, by owners who believed it had been of great service.

There are many patent remedies for this disease before the public; they are all published in the *Official Gazette* of the Patent Office. Some of these remedies contain valuable articles, though not unfrequently the drugs combined are incompatible. A recipe to prevent and to cure, and to be given in all stages and conditions, you would think more likely to do harm than good. Certainly you would have little faith in any specific that might be offered for the prevention and cure of typhoid fever of the human subject, although you would expect to see a larger majority of your patients thus afflicted, recover under treatment properly adapted to their condition.

Mr. President, I am fully aware that the paper I have just read is very unsatisfactory; there are but few points upon which I have been able to speek with absolute certainty. My object will be attained if, in consequence of any discussion that may follow, this disease should hereafter be better understood and more successfully treated.—*Ohio Medical and Surgical Journal.*

